# Supplementary material for: Modeling glioblastoma heterogeneity as a dynamic network of cell states
Source: Mol Syst Biol. 2021 Sep 16;17(9):e10105. doi: 10.15252/msb.202010105 (PMC8444284; doi:10.15252/msb.202010105)
Supplement: Supplementary file 5 — Source Data for Figure 3 [file MSB-17-e10105-s001.zip › Figure3A_sourcedata/GSEA_3065/hallmarks_state5.GseaPreranked.1623416302988/HALLMARK_FATTY_ACID_METABOLISM.html]

Details for gene set HALLMARK\_FATTY\_ACID\_METABOLISM[GSEA]

|  || Dataset | state5 |
| Phenotype | NoPhenotypeAvailable |
| Upregulated in class | na\_neg |
| GeneSet | HALLMARK\_FATTY\_ACID\_METABOLISM |
| Enrichment Score (ES) | -0.47583768 |
| Normalized Enrichment Score (NES) | -1.6406859 |
| Nominal p-value | 0.0012562814 |
| FDR q-value | 0.012505764 |
| FWER p-Value | 0.092 |
Table: GSEA Results Summary

  

Fig 1: Enrichment plot: HALLMARK\_FATTY\_ACID\_METABOLISM      
 Profile of the Running ES Score & Positions of GeneSet Members on the Rank Ordered List

  

| PROBE | GENE SYMBOL | GENE\_TITLE | RANK IN GENE LIST | RANK METRIC SCORE | RUNNING ES | CORE ENRICHMENT || 1 | ERP29 |  |  | 122 | 0.507 | 0.0106 | No |
| 2 | RDH11 |  |  | 224 | 0.422 | 0.0196 | No |
| 3 | PTPRG |  |  | 245 | 0.411 | 0.0371 | No |
| 4 | SERINC1 |  |  | 398 | 0.342 | 0.0364 | No |
| 5 | HIBCH |  |  | 585 | 0.289 | 0.0293 | No |
| 6 | IDH1 |  |  | 793 | 0.242 | 0.0177 | No |
| 7 | DHCR24 |  |  | 795 | 0.242 | 0.0292 | No |
| 8 | OSTC |  |  | 939 | 0.220 | 0.0236 | No |
| 9 | HSD17B11 |  |  | 949 | 0.219 | 0.0331 | No |
| 10 | ELOVL5 |  |  | 1320 | 0.169 | -0.0004 | No |
| 11 | D2HGDH |  |  | 1365 | 0.165 | 0.0026 | No |
| 12 | MLYCD |  |  | 1590 | 0.142 | -0.0158 | No |
| 13 | HMGCS1 |  |  | 1899 | 0.118 | -0.0448 | No |
| 14 | EPHX1 |  |  | 1903 | 0.118 | -0.0395 | No |
| 15 | PPARA |  |  | 2546 | 0.077 | -0.1081 | No |
| 16 | ACOX1 |  |  | 2700 | 0.068 | -0.1221 | No |
| 17 | HSD17B7 |  |  | 3006 | 0.051 | -0.1539 | No |
| 18 | FASN |  |  | 3225 | 0.040 | -0.1766 | No |
| 19 | ADIPOR2 |  |  | 3460 | 0.029 | -0.2015 | No |
| 20 | DLST |  |  | 3524 | 0.026 | -0.2074 | No |
| 21 | NSDHL |  |  | 3526 | 0.026 | -0.2062 | No |
| 22 | GPD2 |  |  | 3594 | 0.024 | -0.2127 | No |
| 23 | HSD17B4 |  |  | 3981 | 0.008 | -0.2558 | No |
| 24 | NCAPH2 |  |  | 4124 | 0.002 | -0.2716 | No |
| 25 | GCDH |  |  | 4320 | -0.007 | -0.2933 | No |
| 26 | SDHA |  |  | 4458 | -0.013 | -0.3081 | No |
| 27 | CPOX |  |  | 4650 | -0.020 | -0.3286 | No |
| 28 | BCKDHB |  |  | 4744 | -0.023 | -0.3380 | No |
| 29 | CPT2 |  |  | 4804 | -0.026 | -0.3434 | No |
| 30 | ACO2 |  |  | 4811 | -0.026 | -0.3428 | No |
| 31 | ACOT8 |  |  | 4827 | -0.027 | -0.3432 | No |
| 32 | ADSL |  |  | 4836 | -0.027 | -0.3428 | No |
| 33 | ACSL1 |  |  | 4862 | -0.028 | -0.3443 | No |
| 34 | BPHL |  |  | 5040 | -0.034 | -0.3626 | No |
| 35 | ALAD |  |  | 5062 | -0.035 | -0.3632 | No |
| 36 | CA2 |  |  | 5139 | -0.038 | -0.3700 | No |
| 37 | METAP1 |  |  | 5180 | -0.039 | -0.3726 | No |
| 38 | HMGCL |  |  | 5270 | -0.043 | -0.3805 | No |
| 39 | G0S2 |  |  | 5284 | -0.043 | -0.3799 | No |
| 40 | GSTZ1 |  |  | 5352 | -0.047 | -0.3852 | No |
| 41 | SUCLG2 |  |  | 5712 | -0.062 | -0.4226 | No |
| 42 | GABARAPL1 |  |  | 5860 | -0.068 | -0.4359 | No |
| 43 | CCDC58 |  |  | 5861 | -0.068 | -0.4327 | No |
| 44 | ENO2 |  |  | 5895 | -0.069 | -0.4330 | No |
| 45 | IDH3G |  |  | 5901 | -0.070 | -0.4303 | No |
| 46 | ALDOA |  |  | 5909 | -0.070 | -0.4277 | No |
| 47 | NBN |  |  | 5977 | -0.073 | -0.4317 | No |
| 48 | ALDH9A1 |  |  | 6006 | -0.074 | -0.4313 | No |
| 49 | SUCLA2 |  |  | 6021 | -0.075 | -0.4293 | No |
| 50 | ACADVL |  |  | 6028 | -0.076 | -0.4263 | No |
| 51 | ACSL4 |  |  | 6041 | -0.076 | -0.4240 | No |
| 52 | RAP1GDS1 |  |  | 6066 | -0.078 | -0.4229 | No |
| 53 | SDHD |  |  | 6111 | -0.080 | -0.4240 | No |
| 54 | HCCS |  |  | 6183 | -0.084 | -0.4280 | No |
| 55 | SDHC |  |  | 6193 | -0.085 | -0.4250 | No |
| 56 | HSDL2 |  |  | 6505 | -0.101 | -0.4551 | No |
| 57 | UROD |  |  | 6565 | -0.106 | -0.4566 | No |
| 58 | UGDH |  |  | 6647 | -0.111 | -0.4605 | No |
| 59 | NTHL1 |  |  | 6763 | -0.118 | -0.4677 | No |
| 60 | HSPH1 |  |  | 6836 | -0.124 | -0.4699 | Yes |
| 61 | IDH3B |  |  | 6850 | -0.125 | -0.4654 | Yes |
| 62 | ACAA1 |  |  | 6891 | -0.128 | -0.4637 | Yes |
| 63 | APEX1 |  |  | 6898 | -0.129 | -0.4582 | Yes |
| 64 | UROS |  |  | 6913 | -0.130 | -0.4535 | Yes |
| 65 | CRAT |  |  | 6922 | -0.131 | -0.4481 | Yes |
| 66 | HSP90AA1 |  |  | 6962 | -0.134 | -0.4460 | Yes |
| 67 | MGLL |  |  | 7100 | -0.145 | -0.4545 | Yes |
| 68 | HADH |  |  | 7129 | -0.148 | -0.4505 | Yes |
| 69 | HADHB |  |  | 7132 | -0.148 | -0.4436 | Yes |
| 70 | PDHA1 |  |  | 7277 | -0.165 | -0.4519 | Yes |
| 71 | IDI1 |  |  | 7337 | -0.172 | -0.4502 | Yes |
| 72 | DLD |  |  | 7363 | -0.176 | -0.4446 | Yes |
| 73 | CBR3 |  |  | 7420 | -0.184 | -0.4421 | Yes |
| 74 | MDH1 |  |  | 7422 | -0.184 | -0.4333 | Yes |
| 75 | ACADM |  |  | 7551 | -0.198 | -0.4382 | Yes |
| 76 | PDHB |  |  | 7586 | -0.202 | -0.4324 | Yes |
| 77 | GLUL |  |  | 7594 | -0.203 | -0.4234 | Yes |
| 78 | ECI2 |  |  | 7776 | -0.229 | -0.4327 | Yes |
| 79 | KMT5A |  |  | 7815 | -0.237 | -0.4256 | Yes |
| 80 | FH |  |  | 7851 | -0.243 | -0.4179 | Yes |
| 81 | GRHPR |  |  | 7895 | -0.255 | -0.4104 | Yes |
| 82 | UBE2L6 |  |  | 7996 | -0.275 | -0.4085 | Yes |
| 83 | ACAA2 |  |  | 8017 | -0.279 | -0.3973 | Yes |
| 84 | DECR1 |  |  | 8034 | -0.282 | -0.3856 | Yes |
| 85 | HSD17B10 |  |  | 8042 | -0.284 | -0.3727 | Yes |
| 86 | ECI1 |  |  | 8099 | -0.297 | -0.3647 | Yes |
| 87 | BLVRA |  |  | 8108 | -0.299 | -0.3513 | Yes |
| 88 | SUCLG1 |  |  | 8202 | -0.327 | -0.3460 | Yes |
| 89 | ECH1 |  |  | 8238 | -0.335 | -0.3339 | Yes |
| 90 | LDHA |  |  | 8263 | -0.343 | -0.3201 | Yes |
| 91 | ALDH3A2 |  |  | 8272 | -0.346 | -0.3044 | Yes |
| 92 | MIF |  |  | 8296 | -0.353 | -0.2900 | Yes |
| 93 | PSME1 |  |  | 8369 | -0.379 | -0.2799 | Yes |
| 94 | ECHS1 |  |  | 8411 | -0.391 | -0.2657 | Yes |
| 95 | PTS |  |  | 8415 | -0.392 | -0.2472 | Yes |
| 96 | ACAT2 |  |  | 8440 | -0.404 | -0.2305 | Yes |
| 97 | CBR1 |  |  | 8480 | -0.424 | -0.2145 | Yes |
| 98 | PCBD1 |  |  | 8533 | -0.448 | -0.1988 | Yes |
| 99 | PRDX6 |  |  | 8673 | -0.540 | -0.1885 | Yes |
| 100 | YWHAH |  |  | 8726 | -0.587 | -0.1661 | Yes |
| 101 | MDH2 |  |  | 8733 | -0.593 | -0.1383 | Yes |
| 102 | SMS |  |  | 8834 | -0.690 | -0.1164 | Yes |
| 103 | ODC1 |  |  | 8839 | -0.697 | -0.0833 | Yes |
| 104 | S100A10 |  |  | 8965 | -0.932 | -0.0526 | Yes |
| 105 | LGALS1 |  |  | 8982 | -1.135 | 0.0002 | Yes |
Table: GSEA details [plain text format]

  

Fig 2: HALLMARK\_FATTY\_ACID\_METABOLISM: Random ES distribution      
 Gene set null distribution of ES for **HALLMARK\_FATTY\_ACID\_METABOLISM**
